# Effect of glucagon-like peptide-1 receptor agonists on adipokine level of nonalcoholic fatty liver disease in rats fed high-fat diet

**DOI:** 10.1515/med-2020-0212

**Published:** 2020-07-18

**Authors:** Miaomiao Jin, Xiaohong Niu, Yan Liu, Dong Zhang, Danni Yuan, Huimin Shen

**Affiliations:** Department of Endocrinology, The Heji Affiliated Hospital of Changzhi Medical College, 271 Taihang East Street, Luzhou District, Changzhi 046011, Shanxi, China; Department of Physiology, Changzhi Medical College, Changzhi 046000, Shanxi, China; Department of Radiology, The Heji Affiliated Hospital of Changzhi Medical College, Changzhi 046011, Shanxi, China; Department of Pathology, The Heji Affiliated Hospital of Changzhi Medical College, Changzhi 046011, Shanxi, China; Department of Laboratory, The Heji Affiliated Hospital of Changzhi Medical College, Changzhi 046011, Shanxi, China

**Keywords:** glucagon-like peptide-1 receptor agonists, adipokine, hepatic steatosis, nonalcoholic fatty liver disease

## Abstract

**Background:**

Nonalcoholic fatty liver disease (NAFLD) is the leading cause of liver disease worldwide, and no effective treatment exists until now. Glucagon-like peptide-1 receptor agonists are becoming the preferred therapeutic option for the management of obesity and are becoming the preferred treatment options for the management of both NAFLD and type 2 diabetes mellitus, but the molecular mechanisms are still unclear.

**Methods:**

Forty-five healthy male Wistar rats were divided into three groups: normal control, high-fat diet (HFD) group, HFD + liraglutide (100 mg/kg body weight) group. Biochemical parameters and adipokine levels were examined in the serum of rats. In order to judge the degree of steatosis of NAFLD, the magnetic resonance imaging and histopathology of the liver were also studied.

**Results and conclusion:**

Liraglutide caused a significant decrease in the serum fasting glucose and improved the insulin resistance, dyslipidemia, and liver enzymes. It reduced the adipokine level, and alleviated the histopathology of liver of rats in the steatosis, ballooning, and lobular inflammation when compared to the HFD group. Thus, liraglutide demonstrated amelioration of NAFLD by decreasing the adipokine levels in this animal model and seems to be a promising molecule for the management of NAFLD.

## 1 Introduction

Nonalcoholic fatty liver disease (NAFLD) is the most common chronic liver disease worldwide [1], affecting approximately 20–35% of the adult population [2]. NAFLD represents a spectrum of diseases ranging from simple steatosis to nonalcoholic steatohepatitis, which may evolve into hepatic fibrosis, cirrhosis, and eventually hepatic carcinoma [3,4,5]. Its pathogenesis is complex and not yet fully understood; however, accumulating data suggest that several adipokines, particularly adiponectin, leptin, resistin, ghrelin, and visfatin, are involved in the manifestation of the disease [6,7,8]. Among these, resistin and visfatin have been intensively studied. Resistin induces hepatic ischemia–reperfusion injury, whereas adipokines are proinflammatory mediators that have been implicated in hepatic lipogenesis and liver fibrogenesis. Pagano et al. reported that serum resistin levels are correlated with the severity of steatosis, inflammation, and fibrosis in patients with NAFLD [6,7]. In addition, visfatin has proinflammatory properties and is increased in patients with ischemia–reperfusion injury [7]. Visfatin levels have been demonstrated to correlate with the severity of hepatic steatosis and fibrosis [8]. However, several previous studies have shown inconsistent results with respect to visfatin level and its correlation with NAFLD [9,10,11]. Previous published reports were mostly human observational studies.

Only limited data exist on the effects of various NAFLD treatment strategies on adipokine levels. Owing to their overall safety and efficacy, glucagon-like peptide-1 receptor agonists (GLP-1RAs) are becoming the preferred therapeutic option for the management of both obesity and type 2 diabetes mellitus (T2DM) [12]. Liraglutide is the most commonly used type of GLP-1RAs. Recent clinical trials have demonstrated that GLP-1RAs could significantly reverse hepatocyte injury and liver inflammation in patients with T2DM and NAFLD [13,14]. However, the effects of GLP-1RAs on NAFLD in obese patients without T2DM are unknown. The precise molecular mechanisms of GLP-1RAs are still unclear. So we inferred that the therapeutic effect of GLP-1RAs on NAFLD was mediated by modulating the adipokine levels.

In this study, we investigated the effects of GLP-1RAs on adipokine levels and hepatocyte steatosis in a rat model of obesity and NAFLD, to help identify the molecular mechanism of GLP-1RAs in reversing hepatocyte injury and liver inflammation in patients with T2DM and NAFLD.

## 2 Materials and methods

### 2.1 Animal model

The study protocol was approved by the Institutional Animal Ethnics Committee of Changzhi Medical College. Forty-five healthy, 6- to 7-week-old male Wistar rats, weighing 120 ± 20 g, were purchased from the Experimental Animal Center of Changzhi Medical College. The rats were housed in a constant environment of 15–25°C with 12-h light/dark cycles. All rats had free access to food and water. The animals were randomly divided into the control group (15 rats) and the model group (30 rats). Rats in the model group were fed a high-fat diet (HFD) (82.5% normal diet + 10% lard + 2% cholesterol; Lanji Technology Development Co., Ltd, Shanghai, China). After 20 weeks, the rats were euthanized and their livers were harvested for pathologic examination to determine whether the NAFLD rat model was successfully established.

### 2.2 Drug intervention

At the end of 12 weeks, 15 rats in the model group were randomly selected and administered with a 0.1 mg/kg/day dose of liraglutide, which was the most commonly used GLP-1RAs (HFD + L group). The remaining 15 rats were administered with sodium chloride physiologic solution (0.9% NaCl) injection (HFD group) for 8 weeks.

### 2.3 Serum biochemical parameters, liver function, adipokines, and hematoxylin–eosin staining of liver tissue

The rats were sacrificed at 20 weeks (15 rats per group). Serum alanine aminotransferase (ALT), aspartate transaminase (AST), triglyceride (TG), total cholesterol (TC), fasting blood glucose (FBG), and fasting insulin (FINS) levels were measured using an automatic biochemical analyzer (Hitachi 7600, Tokyo, Japan). The insulin resistance index (homeostasis model assessment of insulin resistance [HOMA-IR]) was assessed using a steady-state model. HOMA-IR was calculated using the following formula: HOMA-IR = (FBG × FINS)/22.5. The serum resistin and visfatin levels were measured using sensitive enzyme-linked immunosorbent assay kits (EMD Merck Millipore, Burlington, MA, USA; BIO-SWAMP RA20271, Beijing, China). The intra-assay and inter-assay coefficients of variation for resistin and visfatin were <10%.

Liver tissues (1 cm × 1 cm × 0.5 cm) were fixed in 4% multiformaldehyde for 24 h. Thereafter, the tissues were rinsed with water, dehydrated, made transparent, dipped in wax, embedded, and sliced into 5 µm sections for hematoxylin–eosin staining. Histopathologic changes were observed using a microscope, and the NAFLD activity scores (NASs) were computed [15,16].

### 2.4 Hepatic fat fraction of rats measured using in-phase and out-of-phase magnetic resonance imaging

All rats underwent magnetic resonance imaging (MRI) using an axial three-dimensional volumetric interpolated breath-hold examination Dixon sequence, and hepatic fat fraction (HFF) was measured using two regions of interest in the left liver lobe and one region of interest in the right liver lobe. The HFF value was calculated using the following formula: HFF = (SI_in_ − SI_out_)/2 × SI_in_, where SI_in_ means signal of in phase image and SI_out_ means signal of out of phase [17]. The HFF value among different groups of rats and the correlation of HFF value with serum adipokine level were analyzed.

### 2.5 Statistical analysis

SPSS19.0 software (IBM Armonk NY) was used to analyze data. Experimental data are expressed as mean ± standard deviation. One-way analysis of variance was used for in-between group comparisons, and *p* < 0.05 was considered statistically significant. The correlations of serum levels of resistin and visfatin with NASs and HOMA-IR were evaluated using Pearson’s correlation analysis, and partial correlation analysis was performed after adjusting for weight.

## 3 Results

### 3.1 Effect of liraglutide on weight, serum fasting glucose levels, insulin resistance, lipid profile, and liver enzymes

There was a significant increase in weight, serum fasting glucose level, insulin level, and HOMA-IR in rats in the HFD group compared with those in the normal control group (*p* < 0.05) (Table 1). Rats fed HFD showed a significant increase in serum triglyceride, total cholesterol, low-density lipoprotein cholesterol levels (*p* < 0.05), and a significant decrease in high-density lipoprotein cholesterol level (*p* < 0.05), compared with rats in the normal control group (Table 1). Liraglutide (100 mg/kg) significantly reduced the serum glucose level (*p* < 0.05), insulin level (*p* < 0.05), HOMA-IR (*p* < 0.05), and low-density lipoprotein cholesterol level (*p* < 0.05) (Table 1).

**Table 1:** Effect of liraglutide on weight, serum fasting glucose levels, insulin resistance, lipid profiles, and liver enzymes in rats

Variables	NC	HFD	HFD + L
Weight (g)	539.7 ± 61.9	563.7 ± 107.3^*^	534.3 ± 50.1^#^
Fasting glucose (mmol/L)	8.9 ± 0.8	10.4 ± 2.0^**^	8.7 ± 1.0^##^
HOMA-IR	2.84 ± 0.69	12.4 ± 2.1^**^	9.04 ± 2.1^#^
TG (mmol/L)	0.45 ± 0.27	0.85 ± 0.05^*^	0.25 ± 0.09^#^
TC (mmol/L)	2.46 ± 0.37	3.22 ± 0.27^**^	2.0 ± 0.36^##^
LDL-C (mmol/L)	0.51 ± 0.09	0.85 ± 0.02^*^	0.26 ± 0.05^##^
HDL-C (mmol/L)	1.41 ± 0.36	1.39 ± 0.27^*^	1.54 ± 0.08^#^
ALT (IU/L)	23.64 ± 3.16	115.4 ± 5.25^**^	92.94 ± 9.84^#^
AST (IU/L)	27.23 ± 3.59	62.52 ± 5.95^**^	58.6 ± 7.39

Results are shown as mean ± SD (15 rats per group). *p < 0.05 compared to the normal control group,**p < 0.01 compared to the normal control group, ^#^ *p* < 0.05 compared to the HFD group, ^##^ *p* < 0.01 compared to the HFD group. Abbreviations: NC, normal control; HFD, high-fat diet; HFD + L, HFD and treated with 0.1 mg/kg/day liraglutide; HOMA-IR, insulin resistance index; TG, triglycerides; TC, total cholesterol; HDL-C, high-density lipoprotein cholesterol; LDL-C, low-density lipoprotein cholesterol; ALT, alanine aminotransferase; AST, aspartate aminotransferase.

### 3.2 Effect of liraglutide on adipokine levels

HFD induced a significant increase in serum resistin levels compared with the normal diet (*p* < 0.01), whereas liraglutide (100 mg/kg) significantly decreased the resistin levels in HFD rats (*p* < 0.05). Meanwhile, serum visfatin levels showed an increasing trend in the HFD group and a decreasing trend in the HFD + L group, but the difference was not statistically significant (Table 2).

**Table 2:** Effect of liraglutide on serum adipokine levels

Adipokine	NC	HFD	HFD + L
Resistin	0.1 ± 0.02	0.25 ± 0.01^**^	0.20 ± 0.01^##^
Visfatin	1.06 ± 0.1	1.26 ± 0.01	1.15 ± 0.03

Results are shown as mean ± SD (15 rats per group). *p < 0.05 compared to the normal control group, ^**^ *p* < 0.01 compared to the normal control group, ^#^ *p* < 0.05 compared to the HFD group, ^##^ *p* < 0.01 compared to the HFD group. Abbreviations: NC, normal control; HFD, high-fat diet; HFD + L, HFD and treated with 0.1 mg/kg/d liraglutide.

### 3.3 Effect of liraglutide on liver pathology in NAFLD rats

**Figure 1:**
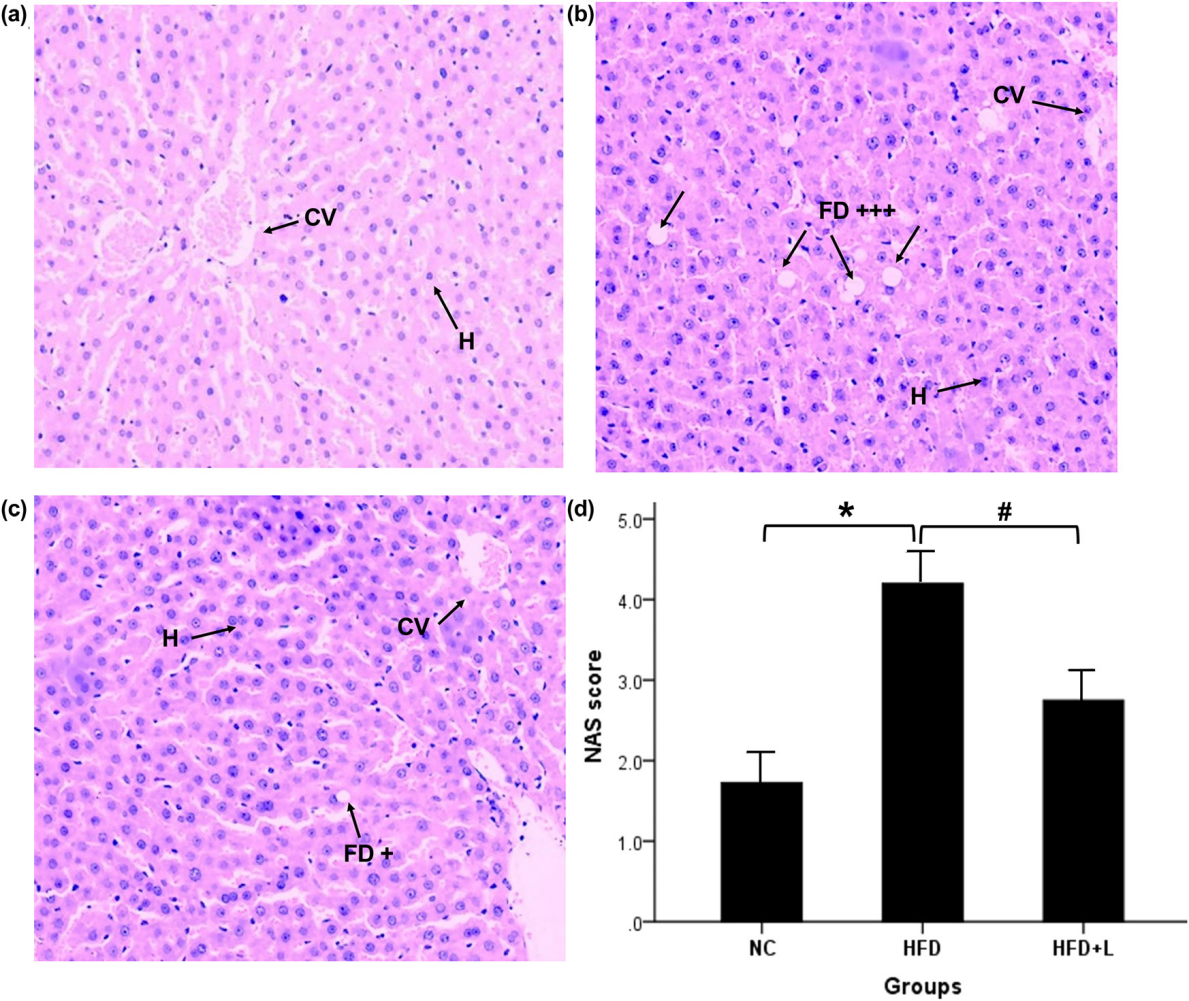
Effect of liraglutide on liver histopathology of rats fed HFD (hematoxylin–eosin staining). Representative images for each group are shown (magnification 100×). (a) Representative liver histopathology of rats in the normal control group (normal hepatocytes and central vein are depicted). (b) Representative liver histopathology of rats fed HFD (severe steatosis, fatty degeneration of hepatocytes, and inflammation are depicted). (c) Representative liver histopathology of rats fed HFD and treated with 100 mg/kg liraglutide (mild fatty degeneration of hepatocytes and moderate inflammation are depicted). (d) NASs of rat livers. Data are shown as mean ± standard deviation for 15 rats per group. *p < 0.05 compared with the normal control group; #*p* < 0.05 compared with the HFD group. Abbreviations: NC, normal control; HFD, high-fat diet; HFD + L, high-fat diet and treatment with 0.1 mg/kg liraglutide; FD, fatty degeneration due to steatosis and ballooning of hepatocytes; CV, central vein; H, hepatocytes; +, mild; +++, severe.

Representative histopathologic images of liver tissues of rats in the different groups are shown in Figure 1. Rats in the normal control group had normal liver architecture with the presence of healthy hepatocytes and central vein (Figure 1a). Livers from rats in the HFD group showed severe fatty deposition, ballooning, and fatty degeneration of hepatocytes with lobular inflammation (Figure 1b). Rats administered with 100 mg/kg liraglutide showed considerable improvement in liver histopathology, demonstrating only mild fatty degeneration of hepatocytes and moderate parenchymatous infiltration of inflammatory cells (Figure 1c). Livers from rats in the HFD group showed higher NASs than the NC group, and administered with 100 mg/kg liraglutide showed considerable lower NASs than the HFD group.

### 3.4 Correlation of adipokine levels with HOMA-IR and NASs

**Figure 2:**
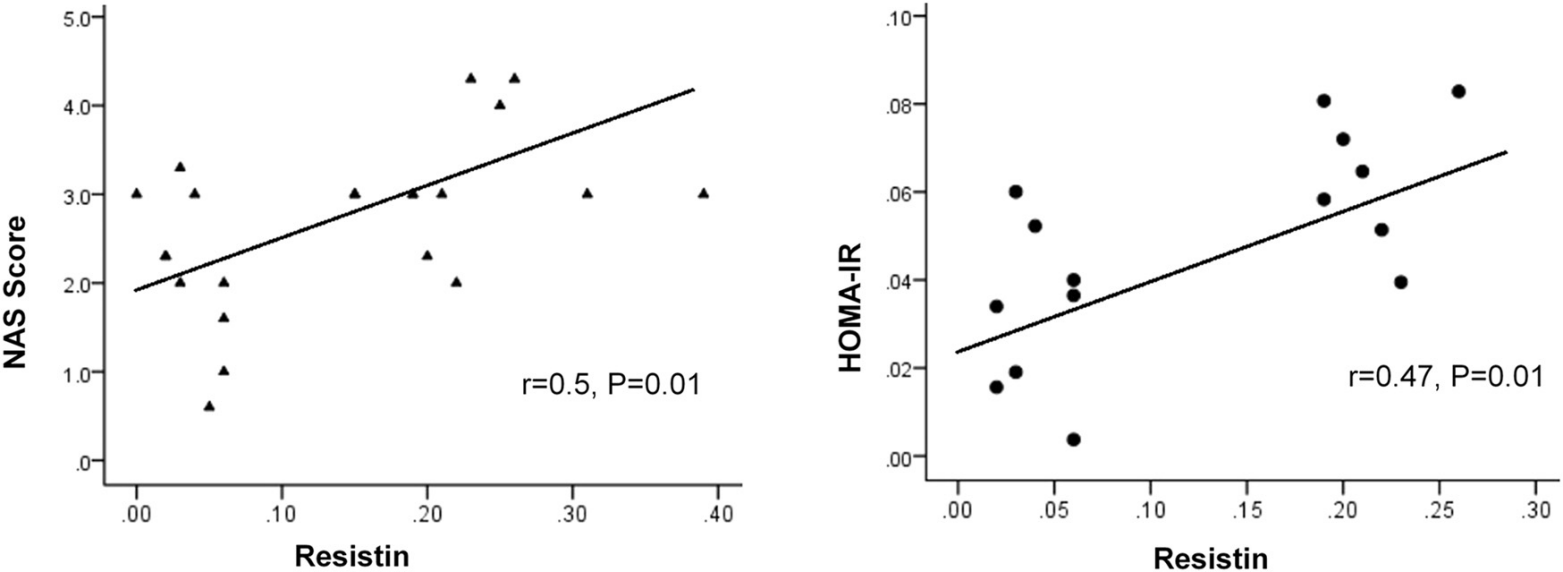
Correlation of serum resistin levels with NASs and HOMA-IR in the rat model of NAFLD. Pearson’s correlation coefficients after adjustment for weight are shown. Serum resistin levels had significant correlations with NASs and HOMA-IR. However, serum visfatin levels had no significant correlations with NASs and HOMA-IR. Abbreviations: NASs, NAFLD activity scores; HOMA-IR, homeostasis model assessment of insulin resistance.

There was a significant positive correlation between resistin levels and HOMA-IR in the HFD and HFD + L groups (*r* = 0.47, *p* = 0.018) (Table 3 and Figure 2). However, no statistical correlation was observed between visfatin levels and HOMA-IR (*r* = 0.20, *p* = 0.51) (Table 3 and Figure 2). There was a significant positive correlation between resistin levels and NASs in rats in the HFD and HFD + L groups (*r* = 0.5, *p* = 0.013) (Table 3 and Figure 2). However, no statistical correlation between visfatin levels and NASs was observed in rats in the fatty liver group (*r* = 0.13, *p* = 0.50) (Table 3).

**Table 3:** Correlation coefficients for serum adipokine levels with NASs and Homa-IR in rats fed high-fat diet

	NASs		Homa-IR	
	Unadjusted	Weight-adjusted	Unadjusted	Weight-adjusted
Resistin	0.50^*^	0.51^*^	0.47^*^	0.51^**^
Visfatin	0.13	0.13	0.20	0.13

Note: Pearson’s correlation coefficients (unadjusted) and partial correlation coefficients (weight adjusted) after adjustment for weight. ^*^ *p* < 0.05, ^**^ *p* < 0.01. Abbreviations: NASs, NAFLD activity scores; HOMA-IR, homeostasis model assessment of insulin resistance.

### 3.5 Correlation of adipokine levels with HFF value measured using MRI

**Figure 3:**
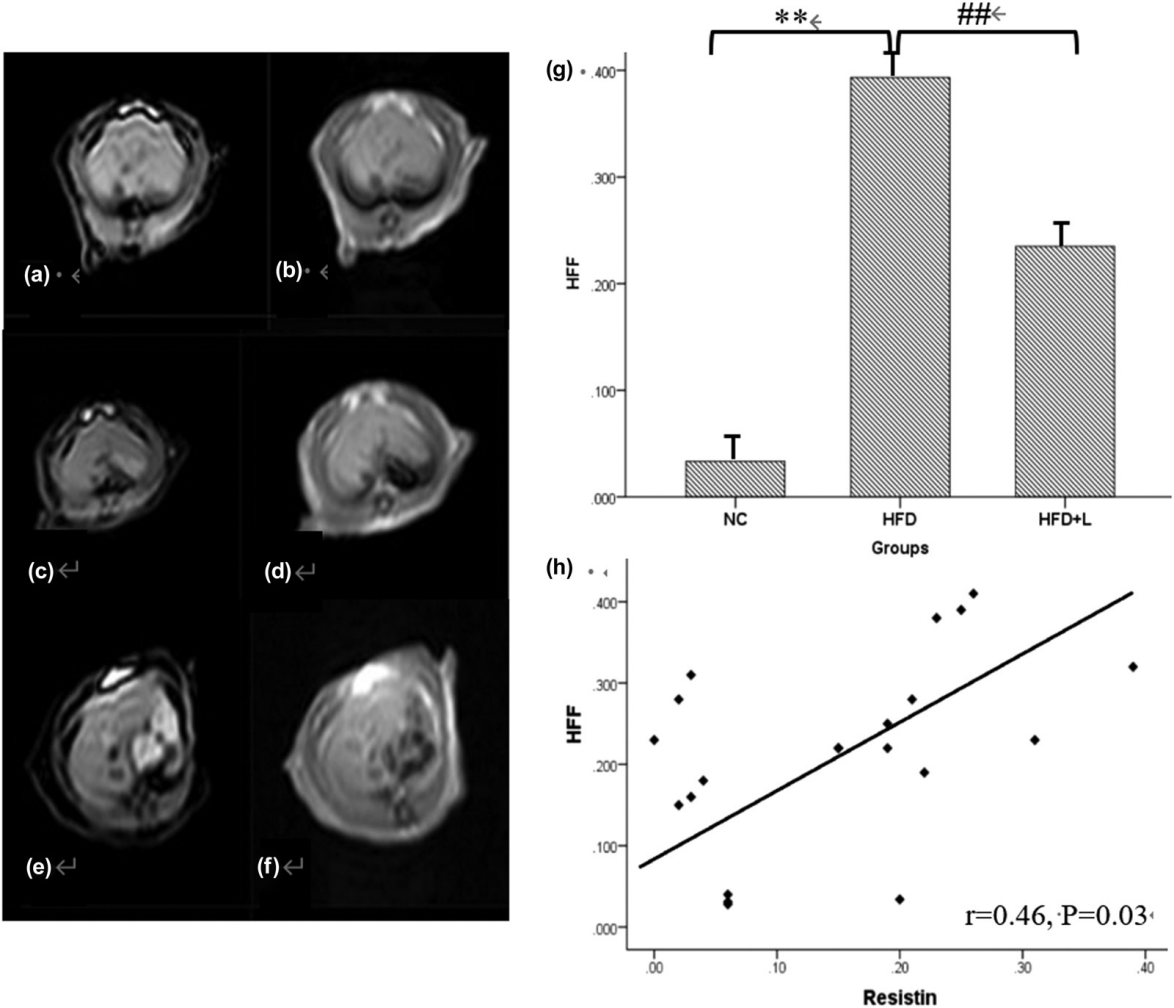
In-phase and out-of-phase magnetic resonance (MR) images and correlation of serum resistin levels with the HFF value in different groups of rats. (a and b) Representative liver in-phase and out-of-phase MR images of rats in the normal control group. The signal of the out-of-phase image was nearly the same as that of the in-phase image. (c and d) Representative liver in-phase and out-of-phase MR images of rats fed an HFD. The out-of-phase image had significantly lower signal than the in-phase image, and the HFF value was 32.23%. (e and f) Representative liver in-phase and out-of-phase MR images of rats fed HFD and treated with 100 mg/kg liraglutide. The out-of-phase image had slightly lower signal than the in-phase image, and the HFF value was 18.67%. (g) HFF value for rat livers of each group. The HFD group have higher HFF value than NC group, and HFD + L group showed considerable lower HFF value than HFD group. Data are shown as mean ± standard deviation for 15 rats per group. **p < 0.01 compared with the normal control group; ^##^ *p* < 0.01 compared with the HFD group. Abbreviations: NC, normal control; HFD, high-fat diet; HFD + L, high-fat diet and treatment with 100 mg/kg liraglutide.(h) Serum resistin level showed a significant positive correlation with the HFF value. However, there was no significant correlation between serum visfatin level and HFF value. Abbreviations: NC, normal control; HFD, high-fat diet; HFD + L, high-fat diet and treatment with 0.1 mg/kg liraglutide; HFF, hepatic fat fraction.

The in-phase and out-of-phase MRI techniques based on the two-point Dixon technique have been confirmed to have high value in diagnosing NAFLD and judging its severity. Using this method, we analyzed liver fatty degeneration and the correlation of the HFF value with serum adipokine level in all rats. As a result, we found a higher HFF value in the HFD group than the NC group (*p* < 0.01), and considerable lower HFF value in the HFD + L group than the HFD group (*p* < 0.01). Moreover, we found a significant positive correlation between resistin levels and HFF value (*r* = 0.47, *p* = 0.03) (Figure 3). However, no statistical correlation was observed between visfatin level and HFF value.

## 4 Discussion

The underlying pathophysiology of NAFLD is not fully understood; however, obesity and insulin resistance seem to play key roles in its manifestation [18,19]. Adipose tissue or body fat is a type of loose connective tissue composed mostly of adipocytes that are derived from preadipocytes. Adipokines, including adiponectin, leptin, resistin, and visfatin, are secreted from adipose tissue [20]. Accumulating data suggest that adipokines are involved in the pathogenesis of NAFLD; hence, they may represent attractive targets for the treatment of patients with NAFLD [21]. However, the available data for circulating adipokines associated with NAFLD remain controversial. Several studies have shown that in patients with NAFLD, serum resistin levels are correlated with insulin resistance and the severity of steatosis, inflammation, and fibrosis [6,7,8]. However, our previous study demonstrated that serum resistin levels were positively correlated with serum lipids and not with insulin resistance in first-degree relatives of patients with T2DM [22]. Li et al. demonstrated that serum visfatin levels were significantly higher in NAFLD rats than in rats in the control group, whereas decreasing visfatin level was therapeutically beneficial [23]. However, the study by Johannsen et al., which included 2,429 human subjects, demonstrated no association between visfatin levels and the presence or absence of NAFLD or the degree of hepatic fatty infiltration [24], whereas Qiu et al. reported that decreased circulating levels of visfatin and adiponectin were independently associated with an increased risk of NAFLD in 372 adults [25]. These inconsistent findings may be related to the different objectives of each study. However, most studies confirmed the role of adipokines in the pathogenesis of NAFLD. In the present study, we also observed a positive correlation of serum resistin level with insulin resistance and NASs in NAFLD rats. Meanwhile, serum visfatin levels showed a tendency toward a positive correlation, but the difference was not statistically significant, which may be due to the insufficient sample size. Our results were consistent with those of the previous studies [6,7,8,23,26]. To further investigate the relationship of adipokines and NAFLD, we used the in-phase and out-of-phase MRI techniques, which have been reported to have a significant correlation with histologic findings of fatty liver, and to have high value in diagnosing and judging the degree of steatosis of NAFLD. As a result, we found a significant positive correlation between serum resistin level and HFF value in rats [17]. This further confirmed the association between resistin level and the degree of hepatic fatty infiltration of NAFLD. Nevertheless, to further verify the results, further studies with a larger sample size are still needed.

Given the role of adipokines in the pathogenesis of NAFLD, interventions aimed at modulating the adipokine levels might have beneficial effects on liver histology. Many pharmacologic agents used in the management of NAFLD affect the adipokine levels. Several studies have shown that pioglitazone, metformin, and vitamin E improve liver histology in NAFLD by affecting the adiponectin levels [27]. Data on the effects of treatments for NAFLD on resistin and visfatin levels are more limited [21]. Although numerous research and clinical studies have demonstrated the efficacy of liraglutide in improving NAFLD [26], its molecular mechanism remains to be elucidated. Improvement in NAFLD after liraglutide administration has been suggested to be associated with the effects of liraglutide on insulin sensitivity and its anti-inflammatory action [28,29,30]. In our study, we used a rat NAFLD model and observed that the serum resistin and visfatin levels in rats in the NAFLD group were significantly higher than those in rats in the control group. The use of GLP-1RAs significantly decreased the serum resistin levels and improved the liver histopathology of rats. In addition, GLP-1RA administration decreased the serum visfatin levels, but the difference was not statistically significant. Our results were consistent with those of the previous studies [6,7,8,23,26]. We believe that GLP-1RAs ameliorate NAFLD by regulating adipokine levels, both systemically and locally, partly independent of weight loss.

This study had some limitations. In the future, we aim to investigate how GLP-1RAs affect the expression levels of adipokines in hepatic tissue and their effect on lipid metabolic genes in the liver.

## 5 Conclusion

Adipokines, particularly resistin and visfatin, seem to play a role in the development of NAFLD in rats fed high-fat diet. Amelioration of NAFLD treated with liraglutide may be mediated by decreasing the resistin and visfatin levels in this animal model. Our study offers additional insights into the potential role of GLP-1RAs as therapeutic agents and of adipokines as therapeutic targets in NAFLD.

## Abbreviations

ALT: alanine aminotransferase
AST: aspartate aminotransferase
FBG: fasting blood glucose
FINS: fasting insulin
GLP-1RAs: glucagon-like peptide-1 receptor agonists
HDL-C: high-density lipoprotein cholesterol
HFD: high-fat diet
HFD + L: high-fat diet and treatment with 0.1 mg/kg liraglutide
HFF: hepatic fat fraction
HOMA-IR: homeostasis model assessment of insulin resistance
LDL-C: low-density lipoprotein cholesterol
MRI: magnetic resonance imaging
NAFLD: nonalcoholic fatty liver disease
NASs: NAFLD activity scores
T2DM: type 2 diabetes mellitus
TC: total cholesterol
TG: triglycerides

## References

[1] Loomba R, Sanyal AJ. The global NAFLD epidemic, nature reviews. Gastroenterol Hepatol. 2013;10:686–90. 10.1038/nrgastro.2013.171 24042449

[2] Chalasani N, Younossi Z, Lavine JE, Diehl AM, Brunt EM, Cusi K, et al. The diagnosis and management of non-alcoholic fatty liver disease: practice guideline by the American Gastroenterological Association, American Association for the Study of Liver Diseases, and American College of Gastroenterology. Gastroenterology. 2012;142:1592–609. 10.1053/j.gastro.2012.04.001 22656328

[3] Ascha MS, Hanouneh IA, Lopez R, Tamimi TA, Feldstein AF, Zein NN. The incidence and risk factors of hepatocellular carcinoma in patients with nonalcoholic steatohepatitis. Hepatology. 2010;51:1972–8. 10.1002/hep.23527 20209604

[4] Wong VW, Vergniol J, Wong GL, Foucher J, Chan HL, Le Bail B, et al. Diagnosis of fibrosis and cirrhosis using liver stiffness measurement in nonalcoholic fatty liver disease. Hepatology. 2010;51:454–62. 10.1002/hep.23312 20101745

[5] Cimini FA, Barchetta I, Carotti S, Bertoccini L, Baroni MG, Vespasiani-Gentilucci U, et al. Relationship between adipose tissue dysfunction, vitamin D deficiency and the pathogenesis of non-alcoholic fatty liver disease. World J Gastroenterol. 2017;23:3407–17. 10.3748/wjg.v23.i19.3407 PMC5442077 28596677

[6] Pagano C, Soardo G, Pilon C, Milocco C, Basan L, Milan G, et al. Increased serum resistin in nonalcoholic fatty liver disease is related to liver disease severity and not to insulin resistance. J Clin Endocrinol Metab. 2006;91:1081–6. 10.1210/jc.2005-1056 16394091

[7] Aller R, de Luis DA, Fernandez L, Calle F, Velayos B, Olcoz JL, et al. Influence of insulin resistance and adipokines in the grade of steatosis of nonalcoholic fatty liver disease. Digest Dis Sci. 2008;53:1088–92. 10.1007/s10620-007-9981-3 17934820

[8] Jamali R, Razavizade M, Arj A, Aarabi MH. Serum adipokines might predict liver histology findings in non-alcoholic fatty liver disease. World J Gastroenterol. 2016;22:5096–103. 10.3748/wjg.v22.i21.5096 PMC4886385 27275102

[9] Dahl TB, Haukeland JW, Yndestad A, Ranheim T, Gladhaug IP, Damas JK, et al. Intracellular nicotinamide phosphoribosyltransferase protects against hepatocyte apoptosis and is down-regulated in nonalcoholic fatty liver disease. J Clin Endocrinol Metab. 2010;95:3039–47. 10.1210/jc.2009-2148 20392873

[10] Akbal E, Kocak E, Tas A, Yuksel E, Koklu S. Visfatin levels in nonalcoholic fatty liver disease. J Clin Lab Anal. 2012;26:115–9. 10.1002/jcla.21491 PMC6807497 22467327

[11] Polyzos SA, Kountouras J, Papatheodorou A, Katsiki E, Patsiaoura K, Zafeiriadou E, et al. Adipocytokines and cytokeratin-18 in patients with nonalcoholic fatty liver disease: Introduction of CHA index. Ann Hepatol. 2013;12:749–57. 24067262

[12] Dhir G, Cusi K. Glucagon like peptide-1 receptor agonists for the management of obesity and non-alcoholic fatty liver disease: a novel therapeutic option. J Investig Med Off Publ Am Feder Clin Res. 2018;66:7–10. 10.1136/jim-2017-000554 28918389

[13] Marso SP, Daniels GH, Brown-Frandsen K, Kristensen P, Mann JF, Nauck MA, et al. Liraglutide and cardiovascular outcomes in type 2 diabetes. N Engl J Med. 2016;375:311–22. 10.1056/NEJMoa1603827 PMC4985288 27295427

[14] Mills EP, Brown KPD, Smith JD, Vang PW, Trotta K. Treating nonalcoholic fatty liver disease in patients with type 2 diabetes mellitus: a review of efficacy and safety. Ther Adv Endocrinol Metab. 2018;9(1):15–28. 10.1177/2042018817741852 PMC5761952 29344336

[15] Song YM, Lee YH, Kim JW, Ham DS, Kang ES, Cha BS, et al. Metformin alleviates hepatosteatosis by restoring SIRT1-mediated autophagy induction via an AMP-activated protein kinase-independent pathway. Autophagy. 2015;11:46–59. 10.4161/15548627.2014.984271 PMC4502778 25484077

[16] Hecht L, Weiss R. Nonalcoholic fatty liver disease and type 2 diabetes in obese children. Curr Diabetes Rep. 2014;14:448. 10.1007/s11892-013-0448-y 24277674

[17] Georgoff P, Thomasson D, Louie A, Fleischman E, Dutcher L, Mani H, et al. Hydrogen-1 MR spectroscopy for measurement and diagnosis of hepatic steatosis. AJR Am J Roentgenol. 2012;199:2–7. 10.2214/AJR.11.7384 PMC3422734 22733887

[18] Younossi Z, Tacke F, Arrese M. Global perspectives on non-alcoholic fatty liver disease and non-alcoholic steatohepatitis. Hepatology. 2019;69:2672–82. 10.1002/hep.30251 30179269

[19] Hashimoto E, Taniai M, Tokushige K. Characteristics and diagnosis of NAFLD/NASH. J Gastroenterol Hepatol. 2013;28(Supp 4):64–70. 10.1111/jgh.12271 24251707

[20] Mousavi Z, Ganji A, Farrokh Tehrani D, Bahari A, EsmaeilZadeh A, Delghandi M. Correlation of visfatin level with non-alcoholic fatty liver in metabolic syndrome. Med J Islamic Repub Iran. 2017;31:28. 10.18869/mjiri.31.28 PMC5804438 29445657

[21] Boutari C, Tziomalos K, Athyros VG. The adipokines in the pathogenesis and treatment of nonalcoholic fatty liver disease. Hippokratia. 2016;20:259–63. PMC5788238 29416297

[22] Niu XH, Li L, Li JY, Song Q, Jin MM, Liu JX. Serum resistin positively correlates with serum lipids, but not with insulin resistance, in first-degree relatives of type-2 diabetes patients: an observational study in China. Medicine. 2017;96:e6622. 10.1097/MD.0000000000006622 PMC5406073 28422857

[23] Li C, Li J, Chen Y, Zhong X, Kang M. Effect of curcumin on visfatin and zinc-alpha2-glycoprotein in a rat model of non-alcoholic fatty liver disease. Acta Cirurgica Brasileira. 2016;31:706–13. 10.1590/S0102-865020160110000001 27982256

[24] Johannsen K, Flechtner-Mors M, Kratzer W, Koenig W, Boehm BO, Schmidberger J. Association between visfatin and hepatic steatosis in the general population during long-term follow-up. Hormone Metab Res. 2019;51:602–7. 10.1055/a-0897-8565 31132798

[25] Qiu Y, Wang SF, Yu C, Chen Q, Jiang R, Pei L, et al. Association of circulating adipsin, visfatin, and adiponectin with nonalcoholic fatty liver disease in adults: a case-control study. Ann Nutr Metabol. 2019;74:44–52. 10.1159/000495215 30541001

[26] Barb D, Portillo-Sanchez P, Cusi K. Pharmacological management of nonalcoholic fatty liver disease. Metabol Clin Exp. 2016;65:1183–95. 10.1016/j.metabol.2016.04.004 27301803

[27] Sanyal AJ, Chalasani N, Kowdley KV, McCullough A, Diehl AM, Bass NM, et al. Pioglitazone, vitamin E, or placebo for nonalcoholic steatohepatitis. N Engl J Med. 2010;362:1675–85. 10.1056/NEJMoa0907929 PMC2928471 20427778

[28] Gupta NA, Mells J, Dunham RM, Grakoui A, Handy J, Saxena NK, et al. Glucagon-like peptide-1 receptor is present on human hepatocytes and has a direct role in decreasing hepatic steatosis in vitro by modulating elements of the insulin signaling pathway. Hepatology. 2010;51:1584–92. 10.1002/hep.23569 PMC2862093 20225248

[29] Rahman K, Liu Y, Kumar P, Smith T, Thorn NE, Farris AB, et al. C/EBP homologous protein modulates liraglutide-mediated attenuation of non-alcoholic steatohepatitis. Lab Invest J Tech Methods Pathol. 2016;96:895–908. 10.1038/labinvest.2016.61 PMC4965279 27239734

[30] Zheng X, Xu F, Liang H, Cao H, Cai M, Xu W, et al. SIRT1/HSF1/HSP pathway is essential for exenatide-alleviated, lipid-induced hepatic endoplasmic reticulum stress. Hepatology. 2017;66:809–24. 10.1002/hep.29238 28439947

